# Molecular Dynamics Study on the Mechanical Properties of Bilayer Silicon Carbide

**DOI:** 10.3390/nano16030207

**Published:** 2026-02-05

**Authors:** Qing Peng, Anyi Huang, Lang Qin, Chaoxi Shu, Jiale Li, Hongyang Li, Lihang Zheng, Xintian Cai, Xiao-Jia Chen

**Affiliations:** 1 School of Science, Harbin Institute of Technology, Shenzhen 518055, China; 2 State Key Laboratory of Nonlinear Mechanics, Institute of Mechanics, Chinese Academy of Sciences, Beijing 100190, China; qinlang25@mails.ucas.ac.cn (L.Q.); 23013080026@stu.hqu.edu.cn (J.L.); hyli@stu.hqu.edu.cn (H.L.); 23014080123@stu.hqu.edu.cn (L.Z.); 3 School of Power and Mechanical Engineering, Wuhan University, Wuhan 430072, China; shu_cx@whu.edu.cn; 4 Key Laboratory of Microgravity (National Microgravity Laboratory), Institute of Mechanics, Chinese Academy of Sciences, Beijing 100190, China; huanganyi25@mails.ucas.ac.cn; 5 School of Engineering Science, University of Chinese Academy of Sciences, Beijing 101400, China; 6 Institute of Manufacturing Engineering, Huaqiao University, Xiamen 361021, China; 7 Institute of Mechanical Engineering and Automation, Huaqiao University, Xiamen 361021, China; 8 School of Mechanical Engineering, Hubei University of Technology, Wuhan 430068, China; 9 Hubei Key Laboratory of Modern Manufacturing Quality Engineering, Wuhan 430068, China

**Keywords:** bilayer silicon carbide, mechanical properties, tensile test, molecular dynamics

## Abstract

The advent of bilayer silicon carbide as a critical two-dimensional material has opened up a range of potential applications in various fields. The field of nanoelectronics and nanomechanical systems is distinguished by its exceptional mechanical robustness, yet the combined effects of environmental and structural factors on its mechanical integrity remain poorly understood. Molecular dynamics simulations are used in this study to systematically examine the tensile response of bilayer SiC across a range of strain rates, temperatures, vacancy concentrations, and pre-existing crack lengths. Results indicate that mechanical properties converge at a system size of 18,144 atoms, ensuring computational efficiency. Increasing strain rate enhances strength and toughness by suppressing atomic relaxation, while elevated temperature induces thermal softening, reducing failure strain and strength by up to 50% at 900 K. Vacancy defects drastically degrade performance, with 3% concentration causing over 70% toughness loss, and crack propagation follows Griffith-type brittle fracture, where the zigzag direction exhibits superior resistance compared to the armchair orientation. These findings highlight the sensitivity of bilayer SiC to defects and environmental conditions, providing critical insights for designing reliable SiC-based nanodevices.

## 1. Introduction

Research on two-dimensional (2D) systems has expanded rapidly in recent years. Their outstanding mechanical, thermal, and electronic characteristics underpin a diverse set of device concepts, including nanoelectronic components, high-temperature sensing elements, flexible electronics, and NEMS platforms [1,2,3,4,5]. Within this materials class, silicon carbide (SiC) is notable as a semiconductor possessing a relatively wide band gap; it combines excellent thermal resilience, a high elastic modulus, pronounced chemical inertness, and strong mechanical robustness, which together make it well suited for demanding applications [6,7,8,9,10,11]. These characteristics make SiC particularly suitable for next-generation devices that must operate under harsh environments where mechanical reliability is a critical concern. While monolayer SiC has been widely studied, emerging evidence suggests that bilayer SiC possesses richer mechanical behavior arising from interlayer coupling, tunable stacking configurations, and enhanced resistance to structural perturbations [12,13,14]. Bilayer structures also more closely resemble realistic device architectures, where multilayer SiC films are routinely integrated into electronic and electromechanical components.

Previous studies have examined the elasticity and fracture behavior of SiC and related 2D materials, yet most investigations have been limited to pristine structures, single-layer membranes, or simplified loading conditions [15,16,17,18,19,20,21,22,23,24,25,26,27,28,29]. A systematic understanding of how system size, temperature, strain rate, vacancy defects, and pre-existing cracks collectively influence the tensile response of bilayer SiC is still lacking. Moreover, the brittle nature of covalent 2D lattices renders their fracture behavior highly sensitive to atomic-scale defects and local stress concentrations [30,31,32,33], emphasizing the importance of a comprehensive mechanical analysis. Nguyen et al. [34] investigated the mechanical response of a family of two-dimensional Si-C sheets using large-scale molecular dynamics and reported direction-dependent elastic and fracture properties for idealized, defect-free structures, emphasizing anisotropy in pristine systems. Le et al. [35] employed atomistic simulations to investigate hexagonal BN and SiC in monolayer form, examining the influence of atomic defects by contrasting defect-free and defect-containing configurations. They found that point defects and vacancies significantly decrease fracture strength and strain, emphasizing the high sensitivity of 2D covalent lattices to atomic-scale flaws. A comprehensive review by Akinwande [36] and co-workers summarized experimental and theoretical advances in 2D mechanics, noting that most reported elasticity and fracture data are derived from pristine or near-pristine samples and that realistic defect-dominated fracture scenarios remain less explored. Recently, Singh et al. [37] systematically elucidated the electronic and structural effects of specific vacancy types in two-dimensional silicon carbide based on first-principles calculations. Their work further confirms that atomic defects profoundly alter local bonding characteristics, thereby influencing mechanical responses.

A detailed assessment of bilayer silicon carbide’s mechanical properties is necessary to determine its applicability in advanced technologies. We used classical molecular dynamics (MD) as the principal tool to examine bilayer SiC under different environmental conditions, specifically focusing on how strain rate and temperature affect plastic deformation and fracture behavior. Furthermore, the mechanical effects induced by structural defects are analyzed through a comparative approach. Our findings enhance knowledge of the intrinsic mechanical behavior of bilayer silicon carbide and yield actionable insights for future applications, with particular relevance to mechanisms of defect-induced failure.

## 2. Materials and Methods

**Figure 1:**
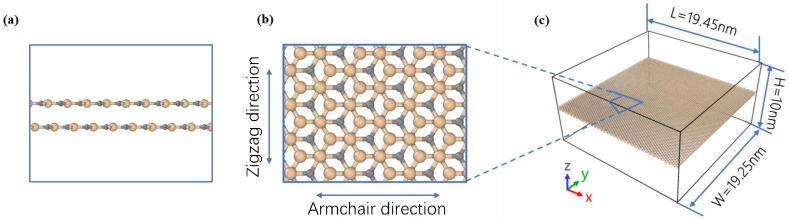
Schematic diagram of model structure. (**a**) View from the front. (**b**) View from above. (**c**) Perspective view.

A double-layer silicon carbide model (Figure 1) was developed; panels (a)–(c) depict its front, top and side views. A Cartesian coordinate framework was defined by aligning the armchair and zigzag directions with the x and y axes, respectively. The most stable bilayer configuration corresponds to AB stacking, for which the lattice constant and interlayer separation are 3.088 Å and 3.234 Å, respectively. In this stacking configuration, silicon atoms of the upper sheet are positioned directly above carbon atoms of the lower sheet, while the upper-layer carbon atoms occupy the vacant registry sites above the lower layer. The structure presented in Figure 1c has dimensions of 19.45 nm by 19.25 nm and consists of 18,144 atoms.

All molecular dynamics simulations were performed on bilayer SiC systems using the LAMMPS package [38]. To model a free-standing bilayer SiC sheet, periodic boundary conditions were applied in the in-plane directions, while a vacuum region was introduced along the out-of-plane direction to eliminate spurious interactions between periodic images. Specifically, the simulation box height was set to 10 nm and the bilayer was placed near the center of the box, so that the minimum image separation along the out-of-plane direction is far larger than the interaction range of the employed short-range potential. Interatomic interactions between Si and C atoms were described by the Tersoff potential [39,40]. The specific Si-C parameterization used in this work is provided in the potential file and summarized in Supplementary Materials, including all pair coefficients and cutoff parameters. We note that alternative parameterizations for SiC exist, as well as emerging machine-learned interatomic potentials. In this work we adopt a classical Tersoff parameterization for computational efficiency and for its suitability in large-scale fracture simulations; a systematic comparison between different potential families is beyond the scope of the present study and will be considered in future work. Time evolution of the atomic system was computed via the velocity Verlet scheme, employing a fixed integration step of 0.001 ps. Before initiating dynamic loading, the atomic configuration was fully relaxed through conjugate-gradient energy minimization to remove residual stresses. Initial thermal conditions were established by assigning atomic velocities randomly along all Cartesian directions. The system was then equilibrated for 40 ps within an NPT ensemble regulated by a Nose-Hoover thermostat to achieve a stable thermodynamic state at the desired temperature and pressure. For uniaxial tensile deformation, stress components transverse to the loading direction were relaxed to maintain zero pressure, while maintaining a fixed temperature condition corresponding to the predefined value during the entire loading stage.

The built-in compute commands in LAMMPS were used to calculate and record stress and strain data, including atomic stress components, average stress, and the von Mises stress. All computational cases, unless explicitly specified, were performed under a temperature of 300 K and a strain rate of 10^9^ s^−1^. Uniaxial tensile testing was performed by progressively expanding the simulation cell along the loading axis, with atomic coordinates remapped at each time step. Stress and strain were recorded during each integration step, with data output at 250-step intervals. Subsequently, the stress–strain relationship was constructed through curve fitting to determine key mechanical property parameters, thereby characterizing the uniaxial tensile response of bilayer silicon carbide under varying conditions. The OVITO package was employed for post-processing tasks, including visualization and atomic structure examination [41].

The mechanical behavior obtained from the simulations was evaluated from multiple perspectives, focusing on strength, deformation, stiffness, and energy absorption. The maximum stress reached during tensile loading was taken as the failure strength, while the strain corresponding to this stress was identified as the fracture strain.Material stiffness was quantified through Young’s modulus, which was extracted from the initial linear elastic regime of the stress–strain response, typically within the first 4% of engineering strain. A vacuum buffer was introduced along the out-of-plane direction to avoid spurious interactions between periodic images. The virial stress reported by LAMMPS is normalized by the total simulation box volume, and thus the presence of a vacuum region would lead to an underestimation of the in-plane stress if used directly. To obtain physically meaningful stresses for the bilayer, we corrected the LAMMPS stress by using an effective thickness for the bilayer SiC, converting the box-volume-normalized stress to a thickness-normalized stress. In this work, the effective thickness was set to a constant value of 7.434 Å, determined from the geometric thickness of the two SiC layers plus the interlayer spacing in the relaxed bilayer structure. For consistency, the same effective thickness was used for all temperatures. In addition, the resistance to fracture was characterized by toughness, defined here as the total mechanical energy absorbed before failure. This quantity was obtained by performing an integration of the stress–strain curve, starting from the undeformed configuration and ending at the fracture point.

## 3. Results and Discussion

### 3.1. Size Effect

Molecular dynamics provides a statistical-mechanics-based route for linking atomistic processes to continuum-level mechanical behavior. In practice, it follows the time evolution of atomic motion and interatomic interactions to extract both microscopic information and macroscopic mechanical properties [42]. However, limited atom numbers in small-scale systems can lead to insufficient statistical sampling and significant fluctuations, whereas large systems dramatically increase computational cost. Accordingly, an appropriate consideration of the size effect is crucial for achieving statistical reliability while maintaining acceptable computational resources.

**Figure 2:**
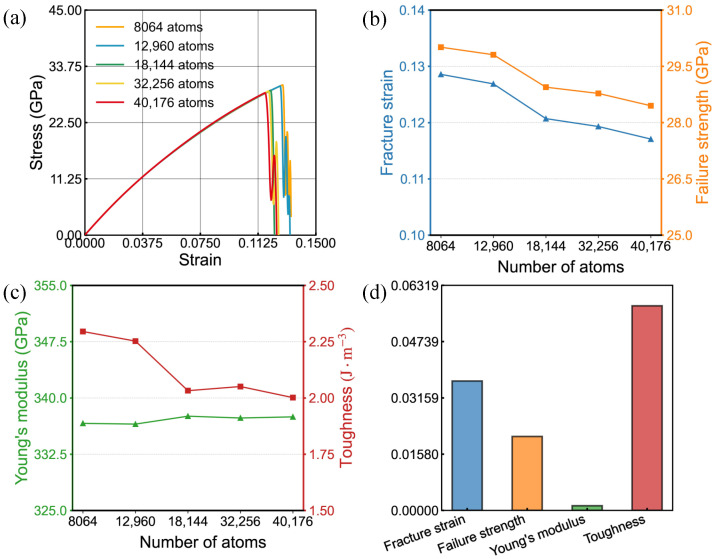
Tensile behavior of bilayer silicon carbide under armchair-oriented loading at various sizes. (**a**) Stress–strain responses. (**b**,**c**) size trends of fracture strain, failure stress, Young’s modulus and toughness. (**d**) Variance in mechanical properties influenced by system dimensions.

**Figure 3:**
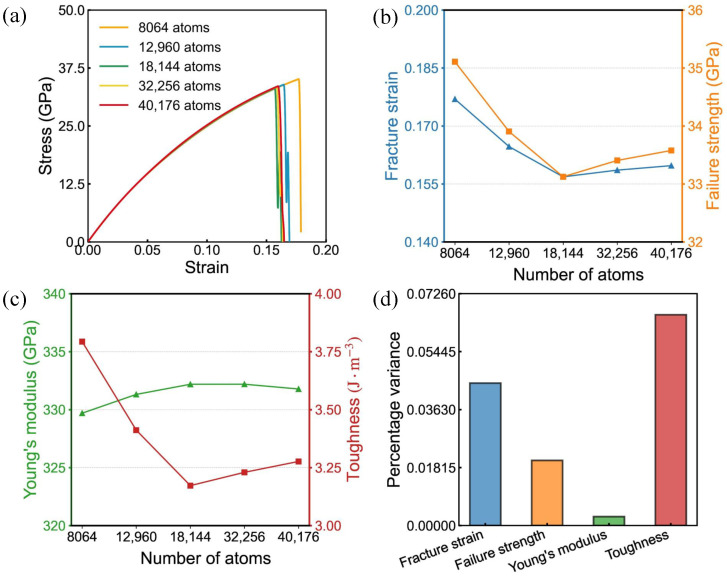
Tensile behavior of bilayer silicon carbide under zigzag-oriented loading at various sizes. (**a**) Stress–strain responses. (**b**,**c**) size trends of fracture strain, failure stress, Young’s modulus and toughness. (**d**) Variance in mechanical properties influenced by system dimensions.

In this work, five defect-free bilayer SiC models with nearly square geometry were constructed, containing 8064 to 40,176 atoms. Uniaxial tensile simulations were first performed along the armchair direction. A separate set of simulations was then carried out along the zigzag direction. Figure 2 and Figure 3 present the computed outcomes. Increasing the system size leads to gradual decreases in failure strength, fracture strain, and toughness, whereas Young’s modulus stays nearly constant. When the atom count exceeds approximately 18,144, the resulting stress–strain curves exhibit near overlap, demonstrating convergence of the simulation results and stabilization of the size effect on the mechanical response. This phenomenon may be attributed to stress wave reflection and statistical fluctuations, with smaller systems potentially exhibiting ’finite-size enhancement’, while larger systems yield results closer to the intrinsic mechanical behavior of the material. To achieve a practical trade-off between accuracy and computational cost, we adopted the 18,144-atom model for the simulations that follow.

To benchmark our results, we compare the pristine bilayer SiC properties with the monolayer MD data reported in Ref. [35], where the mechanical quantities are given in 2D units of N per meter. Using the effective thickness adopted in this work, 7.434 Å, we convert our GPa-based values to 2D units, obtaining a bilayer Young’s modulus of 247 N/m in the zigzag direction and 250 N/m in the armchair direction. Since Ref. [35] reports monolayer values, we further report a per-layer normalized modulus by dividing the bilayer values by two, assuming approximately equal load sharing between the two layers. The resulting per-layer modulus is 123.5 N/m for zigzag and 125.0 N/m for armchair, which are lower than the Ref. [35] values of 179.6 N/m and 173.4 N/m. This difference may arise from differences in the interatomic potential and simulation setup. The ultimate tensile strength is 25.0 N/m along zigzag and 21.6 N/m along armchair, about 22% higher than the Ref. [35] results of 20.5 N/m and 17.6 N/m, whereas the fracture strain, 0.156 and 0.120, is lower than the Ref. [35] values of 0.228 and 0.174 by about 31%. These differences are mainly attributed to the layer number, since a bilayer can provide additional load-bearing pathways, to the thickness convention required when reporting stress in GPa, and to differences in interatomic potential parameterization and loading conditions, while the high strain rates and idealized crystals in molecular dynamics generally elevate strength compared with experiments. The in-plane elastic anisotropy in our bilayer case is weak, within 2% difference, while the failure properties show clearer anisotropy with the zigzag direction exhibiting higher strength and fracture strain.

### 3.2. Strain Rate Effect

**Figure 4:**
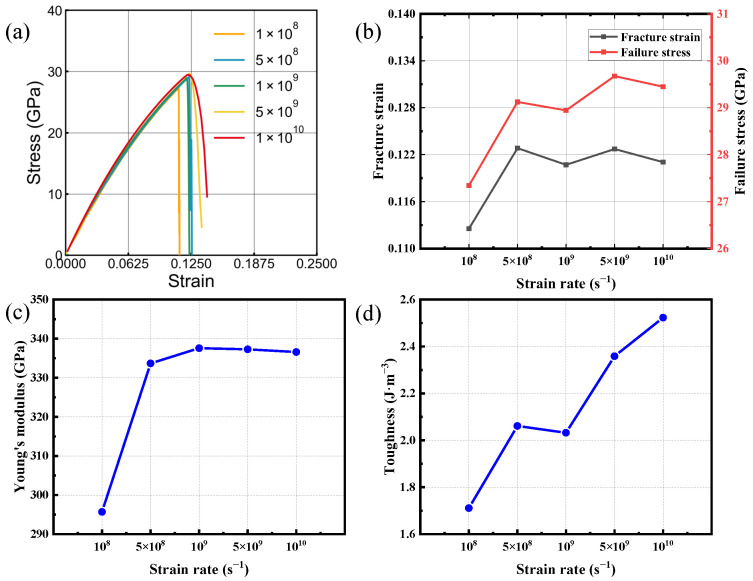
Tensile behavior of bilayer silicon carbide under armchair-oriented loading at various strain rates. (**a**) Stress–strain responses. (**b**–**d**) strain rate trends of fracture strain, failure stress, Young’s modulus and toughness.

**Figure 5:**
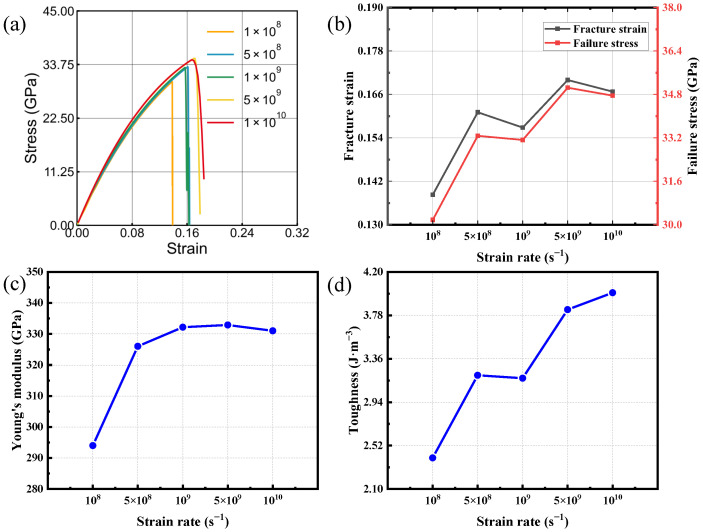
Tensile behavior of bilayer silicon carbide under zigzag-oriented loading at various strain rates. (**a**) Stress–strain responses. (**b**–**d**) strain rate trends of fracture strain, failure stress, Young’s modulus and toughness.

Figure 4 and Figure 5 report uniaxial tensile results for bilayer silicon carbide under armchair loading and under zigzag loading; multiple strain-rate conditions are included. The results indicate that variations in strain rate induce non-monotonic responses in the four mechanical parameters considered. This non-monotonicity is most clearly manifested in the irregular changes observed in fracture toughness and fracture strength (Figure 4b). These variations suggest that multiple competing mechanisms govern the strain-rate-dependent mechanical response of bilayer SiC. Overall, a modest enhancement in Young’s modulus, fracture strain, and failure strength is observed as the strain rate increases. This response arises from the competing effects of atomic relaxation and stress accumulation: at lower strain rates, Si-C bonds and lattice dislocations have sufficient time to diffuse and slip, allowing local stress relaxation and resulting in lower peak strength and toughness. In contrast, at higher strain rates, these relaxation processes are hindered, leading to faster stress buildup and thus higher tensile strength and fracture strain [43,44,45,46]. Based on this analysis, subsequent simulations were carried out using $1\times 10^{9}\;s^{-1}$, as the deviation of mechanical parameters from those at lower strain rates remains within 5%.

### 3.3. Temperature Effect

**Figure 6:**
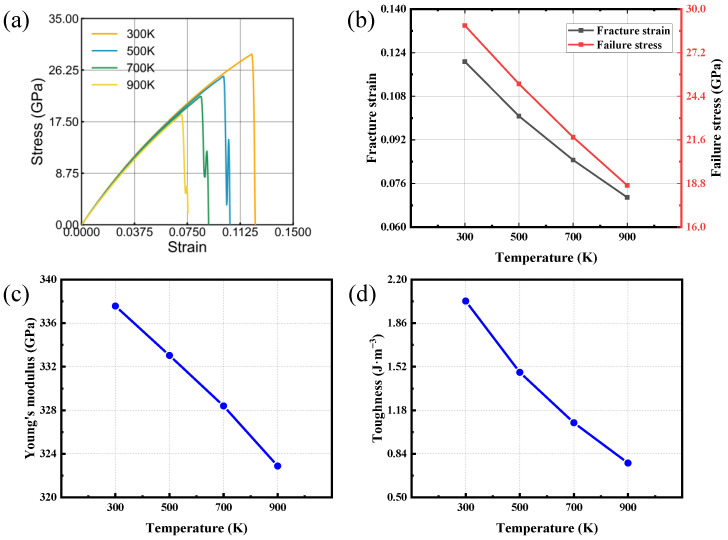
Tensile behavior of bilayer silicon carbide under armchair-oriented loading at various temperatures. (**a**) Stress–strain responses. (**b**–**d**) Temperature trends of fracture strain, failure stress, Young’s modulus and toughness.

**Figure 7:**
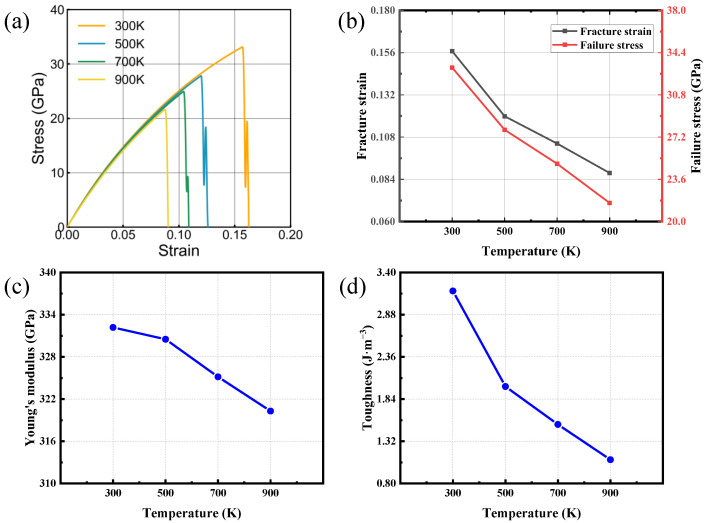
Tensile behavior of bilayer silicon carbide under zigzag-oriented loading at various temperatures. (**a**) Stress–strain responses. (**b**–**d**) Temperature trends of fracture strain, failure stress, Young’s modulus and toughness.

Material mechanical properties are strongly dependent on temperature [47]. To assess temperature-dependent behavior, uniaxial tensile loading was applied to bilayer SiC for two lattice orientations. An engineering deformation rate of 0.001 ${\mathrm{ps}}^{-1}$ was maintained. The temperature was varied from 300 to 900 K. The resulting temperature-dependent trends for the two directions are plotted in Figure 6 and Figure 7. The mechanical performance of bilayer SiC shows a pronounced deterioration at elevated temperatures, manifested by reduced failure strain, lower tensile strength, and diminished fracture toughness. While Young’s modulus exhibits only a slight reduction, demonstrating a typical thermal softening behavior. The mechanical parameters exhibit an approximately linear relationship with temperature, while the stress–strain curves obtained at all investigated temperatures display no discernible plastic deformation stage, thereby indicating the typical brittle fracture behavior of the material.

At lower temperatures, weaker atomic vibrations maintain the stability of Si-C bonds and a compact lattice structure, resulting in higher strength and toughness. In contrast, at elevated temperatures, enhanced thermal vibrations intensify bond-length fluctuations and promote interlayer relaxation, making atoms more likely to reach the critical bond-breaking state [48,49,50]. Consequently, both strength and toughness are significantly reduced with increasing temperature. Comparison between the two loading directions reveals that the zigzag direction consistently exhibits higher fracture strain, failure strength, and fracture toughness than the armchair direction, suggesting that its atomic configuration and bonding geometry are more favorable for stress distribution and load transfer under tension. In summary, increasing temperature leads to pronounced mechanical degradation of bilayer SiC in both orientations, while the zigzag direction shows superior resistance to thermal disturbance and better mechanical stability.

### 3.4. The Vacancy-Defect Effect

In crystalline materials, a vacancy corresponds to an unoccupied lattice site where an atom or ion would normally reside, and it is widely recognized as a common point defect. Such defects can significantly influence mechanical behavior, often promoting brittle fracture and reducing fatigue resistance during processing and service [51,52,53,54,55,56,57,58,59]. From an engineering perspective, controlling the formation and distribution of vacancies is an effective approach to tailoring material properties. In semiconductor systems, vacancy regulation is particularly critical, as it can alter the electronic structure and thereby improve device performance. Therefore, vacancy behavior is a key topic for materials-oriented fundamentals and for engineering use, and it directly supports reliability evaluation in real service environments. To probe vacancy influence, atoms were removed at random (0.1%, 0.3%, 0.5%, 1% and 3%) from a simulation cell containing 18,144 atoms, and the resulting mechanical response of bilayer SiC was evaluated. Tensile simulations were then carried out on the defective structures, with the corresponding responses benchmarked against a defect-free bilayer reference.

**Figure 8:**
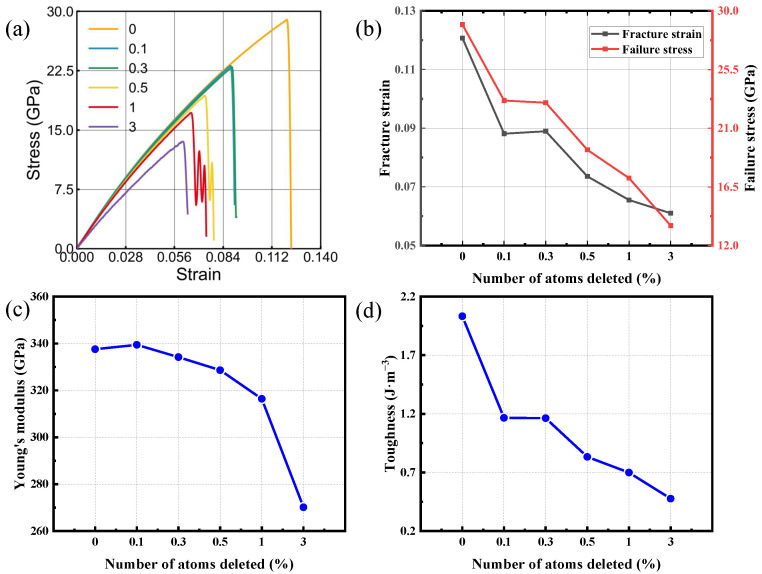
Tensile behavior of bilayer silicon carbide loaded in the armchair orientation with systematically varied vacancy content. The curves in (**a**) illustrate the tensile response for each defect level. Panels (**b**,**d**) quantify defect-content trends in fracture strain and failure stress (**b**), elastic modulus (**c**), and energy absorption capability (toughness) (**d**).

**Figure 9:**
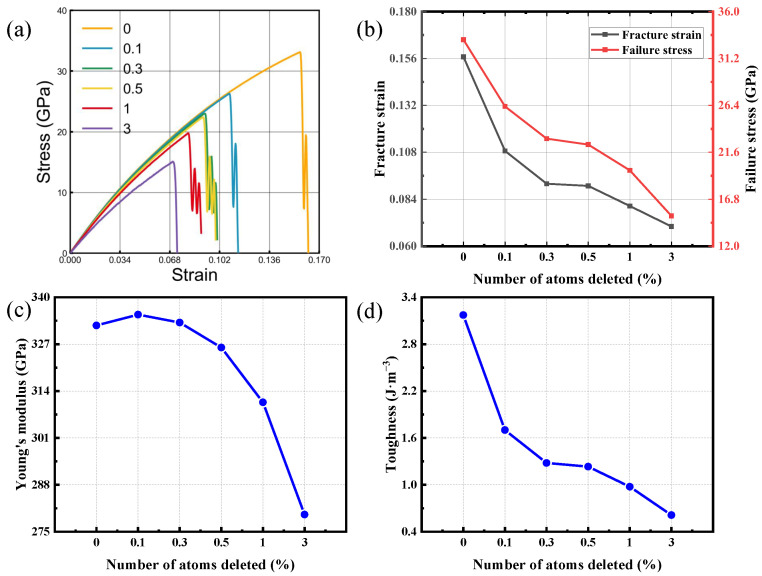
Tensile behavior of bilayer silicon carbide loaded in the zigzag orientation with systematically varied vacancy content. The curves in (**a**) illustrate the tensile response for each defect level. The remaining panels quantify defect-content trends in fracture strain and failure stress (**b**), elastic modulus (**c**), and energy absorption capability (toughness) (**d**).

Figure 8 and Figure 9 present the tensile mechanical response of bilayer SiC when loaded along the armchair orientation and, separately, along the zigzag orientation. The corresponding variations in four mechanical parameters are further presented for different vacancy concentrations. Elevated vacancy levels lead to a marked deterioration in the mechanical performance of the system, manifested by reduced fracture strain, lower failure strength, diminished Young’s modulus, and weakened fracture toughness. Notably, this deterioration becomes much more severe when the defect content rises beyond approximately 1%. When loaded along the armchair orientation, the material shows a marked decline in failure strain—from roughly 12% to 6%—as vacancies become more abundant, and the tensile strength drops from 29 GPa to 13 GPa, the toughness decreases by more than 70%, and Young’s modulus is reduced by nearly 20%. These results indicate that vacancies strongly weaken both the load-bearing capacity and deformation resistance of the bilayer structure. Although the overall mechanical performance under zigzag directional loading is superior to that under armchair directional loading, its performance still deteriorates significantly as the void concentration increases; even a minute vacancy level of 0.1% can reduce the strength and toughness by approximately 20% and 40%, respectively, demonstrating the high sensitivity of bilayer SiC to defect formation.

This degradation may stem from the disturbance caused by atomic vacancies to the local lattice and bonding network. The absence of Si or C atoms reduces the bond density and bond strength around the defect sites, leading to stress concentration and facilitating the initiation and propagation of cracks. Meanwhile, the defect-induced local relaxation and bond distortion diminish the uniformity of atomic interactions, thereby increasing the susceptibility of the material to brittle failure when subjected to external loading. As the vacancy concentration further increases, these localized stress concentration effects accumulate, resulting in a simultaneous reduction in stiffness, strength, and toughness. Overall, vacancies act as precursors to microcracks, disrupting the structural continuity and cooperative deformation of bilayer SiC. Consequently, both crystallographic directions exhibit pronounced brittleness and strength degradation, while the zigzag direction, due to its denser atomic packing, shows slightly higher resistance to defect-induced weakening and better mechanical stability.

### 3.5. Cracks and Toughness

Crack defects already present within the material strongly affect its mechanical response. In practical engineering and service environments, fracture rarely initiates from the direct breaking of atomic bonds in a perfect lattice; instead, it typically originates from the propagation of microcracks that already exist within the material. Consequently, variations in the size, shape, and spatial distribution of pre-existing cracks critically affect a material’s resistance to failure.

According to the classical Griffith fracture criterion [60],

(1) $$\sigma_{f}=\sqrt{\frac{2E\gamma }{\pi a}}$$

In this formulation, $\sigma_{f}$ is used to indicate the stress at fracture, while γ quantifies the associated surface energy. Young’s modulus *E* is used as a measure of elastic stiffness, and the crack size is characterized by the parameter *a*. Owing to its effectiveness, this model has been widely employed to describe fracture processes in brittle solids, including ceramics, glass, and some polymers, and it serves as an important theoretical basis for the design of advanced materials and structural components aimed at reducing brittle failure.

**Figure 10:**
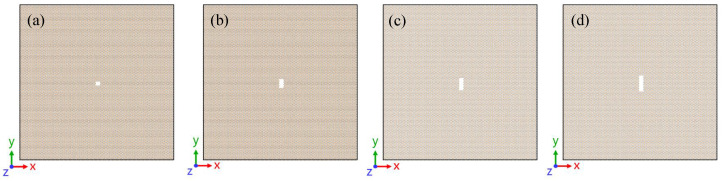
Bilayer silicon carbide model with cracks of varying lengths. (**a**) 0.5 nm crack; (**b**) 1.0 nm crack; (**c**) 1.5 nm crack; (**d**) 2.0 nm crack.

Failure strength depends sensitively on the surface energy as well as the characteristic size of the crack. When the localized stress field near the crack front becomes sufficiently large to break one or more atomic bonds, the crack becomes unstable and propagates spontaneously. The influence of crack defects on bilayer SiC was investigated by embedding central rectangular cracks of varying dimensions within the model. In this study, through-thickness cracks were considered, meaning that the crack penetrates completely from one surface to the opposite surface of the material. Such cracks are generally more hazardous than surface cracks because they can more effectively compromise the load-bearing capability. Four crack sizes were considered in this study, ranging from sub-nanometer to nanometer scale (0.5–2 nm), with their geometric configurations shown in Figure 10.

#### 3.5.1. Effect of Cracks on Mechanical Properties

**Figure 11:**
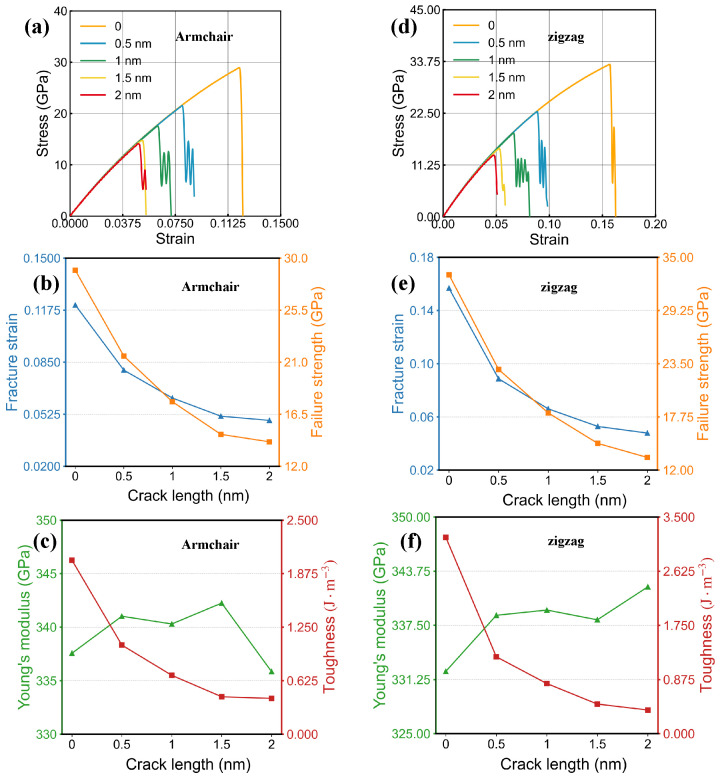
Tensile responses of bilayer SiC models containing centrally positioned rectangular cracks with different lengths. Panels (**a**–**c**) correspond to loading applied along the armchair-oriented lattice: (**a**) Stress–strain curves. (**b**,**c**) crack-length-dependent evolutions of four representative mechanical parameters. Panels (**d**–**f**) present the corresponding results for loading applied along the zigzag-oriented lattice. (**d**) Stress–strain curves. (**e**,**f**) crack-length-dependent changes in the same set of mechanical parameters.

Figure 11 provides an overview of tensile behavior in bilayer SiC models that include centrally positioned cracks with varied dimensions, for loading applied along the armchair and zigzag lattice orientations. The data reveal that increasing crack dimension leads to substantial losses in strain-at-failure, strength, and toughness; in contrast, the elastic modulus undergoes only minor variation, indicating limited sensitivity of stiffness to crack scale.

In the armchair direction, extended cracks markedly impair the overall mechanical performance, leading to characteristic brittle fracture behavior. The relationship between performance and crack length exhibits a decreasing trend: the reduction in failure strain, strength, and toughness is most rapid when the crack is initially introduced, but the slope gradually decreases as the crack extends further. This trend suggests that the material is most vulnerable during the crack initiation stage, where strong stress concentrations develop at the crack tip and accelerate localized bond breaking. Once the crack grows beyond the initial stage, the associated stress field becomes more stable, making the incremental impact on global stiffness and strength comparatively smaller. The stress–strain curves nearly overlap in the elastic region for all crack lengths, further indicating that the elastic response is insensitive to cracking and that fracture is predominantly governed by crack-tip instability. Similar trends are observed for the zigzag direction. Increasing crack length again causes substantial reductions in failure strain, strength, and toughness, while the Young’s modulus remains nearly constant. The diminishing-decline trend is also present, reaffirming that damage is concentrated near the crack tip, whereas the overall lattice rigidity remains largely intact. Compared with the armchair direction, however, the zigzag orientation consistently exhibits higher strength and toughness across all crack lengths. This indicates that its atomic bonding configuration provides a more favorable stress distribution and renders the material less sensitive to cracking, thereby enhancing its resistance to crack-induced failure.

In the Griffith-type analysis, the crack length *a* denotes the half-length of a central crack; for the rectangular cracks discussed below, the reported crack length *L* corresponds to the full crack length, and thus a=L/2 is used when examining Griffith scaling. Although Equation (1) gives the Griffith fracture criterion, we now explicitly examine whether our crack simulations follow the expected scaling. For brittle fracture governed by Griffith-type behavior, the fracture strength is predicted to decrease approximately in proportion to $1/\sqrt{a}$. Using the fracture strengths obtained for different crack lengths, we find that $\sigma_{f}$ exhibits an approximately linear dependence on $1/\sqrt{a}$ over the investigated range from 0.5 to 2.0 nm. A linear fit yields a high goodness-of-fit with $R^{2}\approx 0.989$ for both armchair and zigzag loading, and the maximum deviation among data points is within about 4%, indicating that the observed crack-length dependence is consistent with Griffith-type scaling at the nanoscale. Based on Equation (1), we further back-calculate an effective surface energy from the simulated $\sigma_{f}\left(a\right)$ data combined with the corresponding Young’s modulus, obtaining $\gamma_{\mathrm{eff}}\approx 1.60\;J/m^{2}$. Finally, using the experimentally reported tensile strength range of 3–5 GPa, the corresponding critical crack length for catastrophic fracture is estimated to be on the order of 14–38 nm, highlighting the strong sensitivity of measured strength to the presence of nanoscale flaws.

Overall, crack extension markedly accelerates the brittle failure of bilayer SiC but has limited influence on its elastic modulus. Meanwhile, a pronounced directional dependence is observed. The zigzag direction exhibits superior mechanical stability, which offers valuable guidance for the design of micro- and nanoscale SiC structures with enhanced load-bearing capability.

#### 3.5.2. Deformation and Fracture Behavior

**Figure 12:**
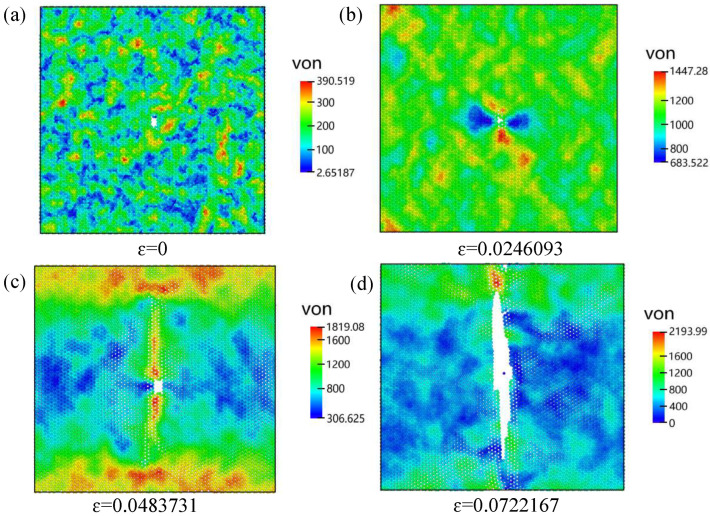
Von Mises stress distribution in uniaxially stretched bilayer silicon carbide containing a 1-nanometer-long rectangular crack. (**a**) ε=0; (**b**) ε=0.0246; (**c**) ε=0.0484; (**d**) ε=0.0722.

Figure 12 illustrates the evolution of the equivalent stress field during armchair-direction tensile loading for a bilayer SiC model containing a 1 nm central crack. Overall, the results indicate a progressive intensification of localized stress near the crack front as strain increases. This behavior ultimately governs crack propagation and final structural failure.

In the initial relaxed state (Figure 12a), the system exhibits a low and nearly uniform stress distribution, indicating a stable lattice configuration without noticeable local concentration. As tensile strain is applied (Figure 12b), the global stress level rises and pronounced concentration first appears at the crack tips, displaying a typical Mode-I opening pattern. This indicates that the crack-tip region has already become the most susceptible site for instability.

With further tensile loading approaching the failure stage (Figure 12c), the upper part of the crack begins to extend, accompanied by a sharp increase in stress around the crack tip. The stress field becomes markedly asymmetric, suggesting that the system has entered an unstable crack-growth regime in which the local stress maximum dictates the subsequent fracture path.

At final failure (Figure 12d), the cracks in the two layers propagate and coalesce into a fully developed through-thickness fracture surface. Stress is rapidly released along the fracture path, characteristic of a brittle failure mode. This evolution clearly demonstrates that the continuous intensification of crack-tip stress concentration is the primary driving mechanism for crack propagation, and thus serves as a critical indicator for predicting fracture behavior and assessing the mechanical reliability of bilayer SiC.

## 4. Conclusions

This work examines how system size, temperature, loading rate, vacancy defects, and crack-related features affect the tensile response of bilayer silicon carbide. It is found that larger systems exhibit progressive decreases in failure strain, tensile load-bearing capacity, and energy absorption, whereas the Young’s modulus remains nearly unchanged. Mechanical properties converge once the system reaches approximately 18,144 atoms, indicating that this scale provides a reasonable balance between statistical accuracy and computational efficiency. Temperature was found to strongly influence the material’s strength and deformation capacity: from 300 to 900 K, the failure strain, tensile strength, and toughness decrease almost linearly, whereas the Young’s modulus exhibits only a slight reduction. The observed softening originates from temperature-driven atomic motion, which reduces the strength of Si-C bonds and facilitates relaxation between layers. In contrast, increasing the strain rate stiffens the material response. Higher loading rates suppress dislocation relaxation and inhibit early crack initiation, thereby enhancing Young’s modulus and toughness while simultaneously increasing the overall strength and failure strain.

Structural imperfections impose a more pronounced deterioration in mechanical performance. Even a very low vacancy concentration of 0.1% can reduce strength and toughness by more than 20%, and at 3% vacancy concentration, toughness in both directions drops by over 75%. Vacancies act as embryonic microcracks, inducing severe local stress concentrations and accelerating brittle failure. Pre-existing cracks further amplify this degradation: when the crack length increases from 0 to 2 nm, the failure strain, strength, and toughness each decrease by more than half, accompanied by a diminishing rate of degradation at larger crack lengths. In this regime, localized stress near the crack front governs the brittle fracture process. In addition, the zigzag orientation consistently exhibits higher strength and toughness than the armchair orientation, underscoring the intrinsic mechanical anisotropy of bilayer SiC. This directional trend is consistent with molecular dynamics results widely reported for other hexagonal two-dimensional materials [35]. For example, representative simulations on graphene and h-BN show higher Young’s modulus and tensile strength along the zigzag direction than along the armchair direction, and similar anisotropic behavior has also been reported for two-dimensional SiC. These comparisons indicate that the zigzag-dominant strength observed in our bilayer SiC is not an isolated outcome, but rather aligns with the common anisotropy of covalently bonded two-dimensional lattices.

In summary, this study systematically elucidates the key factors influencing the mechanical stability of bilayer SiC and reveals the underlying microscopic mechanisms associated with its brittle fracture. These results clarify how loading conditions and representative defect states influence the tensile failure of bilayer SiC, providing mechanistic insight that can inform reliability assessment of SiC-based nanoelectronic and NEMS structures.

## Data Availability

The datasets generated during and/or analyzed during the current study are available from the corresponding author on reasonable request.

## Supplementary Materials

The following supporting information can be downloaded at https://www.mdpi.com/article/10.3390/nano16030207/s1, Figure S1: Fracture strength versus inverse square root of crack length for bilayer SiC under armchair and zigzag loading.
