# Supplementary figures and images for: Molecular Dynamics Study on the Mechanical Properties of Bilayer Silicon Carbide
Source: Nanomaterials (Basel). 2026 Feb 5;16(3):207. doi: 10.3390/nano16030207 (PMC12899606; doi:10.3390/nano16030207)

# Griffith-type scaling: bilayer SiC

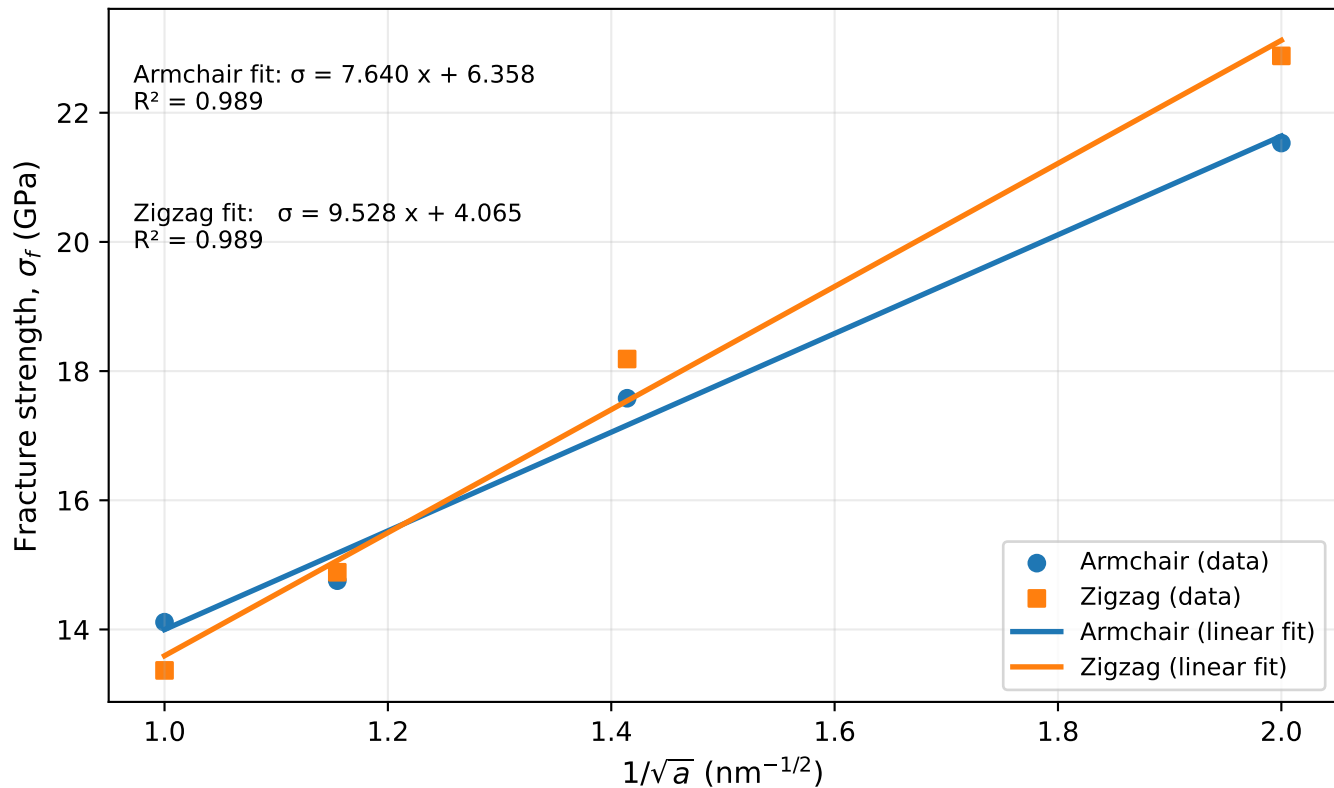

Supplement: Supplementary file 1 [file nanomaterials-16-00207-s001.zip › Figure_S1_Griffith_scaling.pdf]
